# A lesion of the patella: An unexpected location of Rosai-Dorfman disease: A case report

**DOI:** 10.1016/j.ijscr.2022.107510

**Published:** 2022-08-13

**Authors:** Farah Sassi, Haythem M'rad, Linda Belhaj Kacem, Boubaker Sassi, Samia Hannachi, Soumaya Rammeh

**Affiliations:** a Pathology Department, Charles Nicolle Hospital, Tunis, Tunisia; b Orthopedic Department, Centre de Traumatologie et des Grands Brûlés, Tunis, Tunisia; c Orthopedic Department, Centre Urbain Nord, Tunis, Tunisia; d Pathology Laboratory, Centre Urbain Nord, Tunis, Tunisia

**Keywords:** Rosai–Dorfman disease, Bone involvement, Extranodal involvement, Patella, Case report

## Abstract

**Introduction:**

Primary osseous Rosai-Dorfman disease (RDD) is a rare and benign disease that can pose diagnosis challenges.

**Presentation of the case:**

We report the case of a 29-year-old woman who presented with pain in her left patellar region for the past 6 months with no other clinical sign especially no lymphadenopathy associated. A surgical excision was done. Histopathology confirmed the diagnosis of a primary RDD patellar disease. The patient was followed up for 2 months without any recurrence. The aim of this study was to present a rare case of RDD in a patellar location and to review clinicopathological features, therapeutic modalities, evolutionary aspects and prognosis of a primary patellar RDD.

**Discussion and conclusion:**

The diagnosis of a primary osseous RDD without associated lymphadenopathy should be kept in mind when a sclero-lytic lesion is found. Excision of the lesion is the gold standard of the treatment.

## Highlights

• Isolated patellar RDD is rare.
• The diagnosis is made on histopathological examination of resected tissue
• Prognosis is good and disease course is not aggressive

## 1 Introduction

Rosai-Dorfman disease (RDD) is a rare and benign disease also known as sinus histiocytosis with massive lymphadenopathy typically presenting as massive bilateral cervical lymphadenopathy [1], [2]. Extranodal RDD's involvement is common and occur in >40 % of patients. The most frequent extranodal RDD sites include the skin (10 %) [3], nasal cavity (11 %) [4], orbital tissue (11 %) [4] and central nervous system (<5 %) [4]. Bone involvement is seen in 5 % to 10 % of extranodal sites with usually multifocal lesions with concomitant lymphadenopathy [4], [5]. Solitary lesions of bone without nodal involvement or additional clinical features are exceedingly rare; only a few case reports have been reported in the literature, as well as a large series describing 15 cases [6]. Most often osseous lesions are seen in the metaphysis and in craniofacial skeleton. Solitary patellar RDD involvement is extremely rare and has been reported in one case [7]. Here, we present the second case, to the best of our research, of RDD arising in the patella.

The aim of the study was to discuss clinical, radiological and pathological features with emphasis on differential diagnosis of patellar RDD.

## 2 Case presentation

**Fig. 1:**
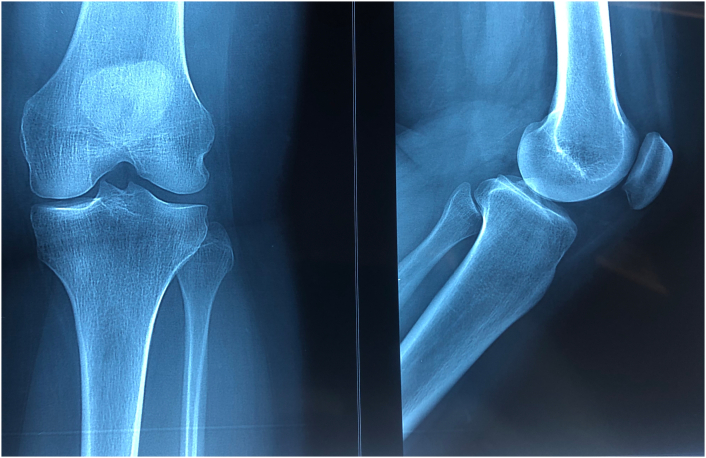
Standard radiograph showing nothing abnormal.

**Fig. 2:**
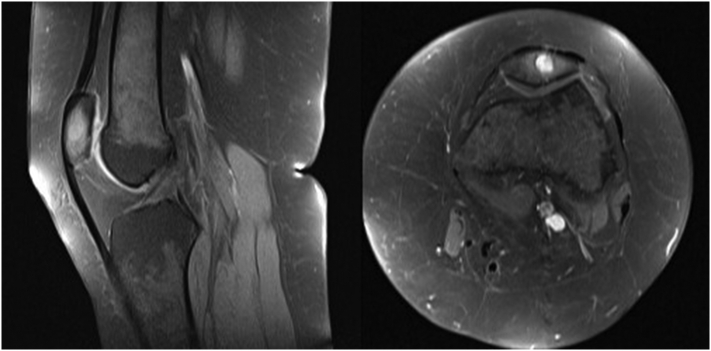
MRI T1-weighted with fat saturation coronal and axial sections showing hyperintense well defined lesion in the left patellar.

**Fig. 3:**
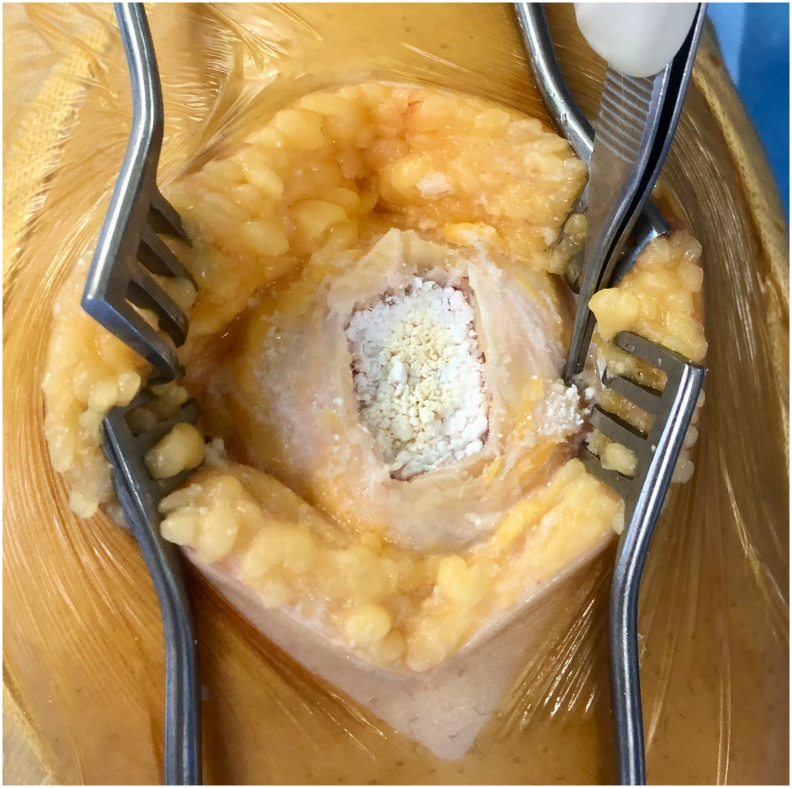
Intraoperative photo after curettage and using granular substitute bone.

**Fig. 4:**
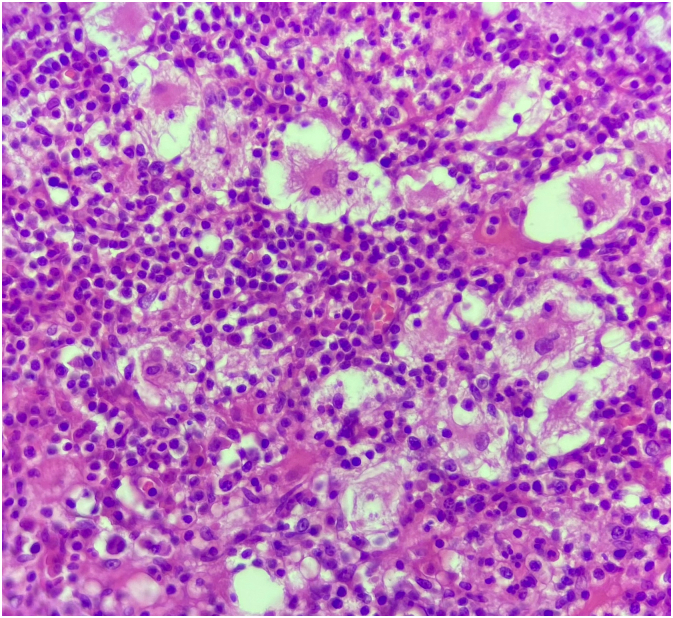
Pathologic examination section showing bony trabecula surrounded by numerous foamy enlarged histiocytes composed with round to oval hypochromatic nuclei and abundant eosinophilic cytoplasm, often containing engulfed inflammatory cells known as emperipolesis, admixed with a dense inflammatory mononuclear cells comprising lymphocytes, neutrophils, and scattered eosinophils and plasma cells (H&E. ×40).

**Fig. 5:**
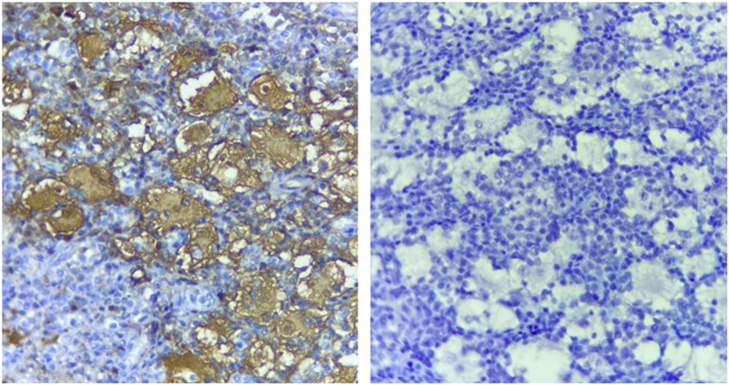
Immunohistochemistry showing positivity for S-100 protein and negativity for CD-1a.

A 29-year-old morbidly obese gravida 2, para 2 woman with unremarkable past medical history presented with pain in her left patellar region since 6 months with no other clinical sign which was partially managed with analgesic medication. Cell blood count (CBC), ultrasound and conventional radiograph of her knee were normal (Fig. 1). Right knee magnetic resonance imaging (MRI) exhibited an 8 mm well-defined round lesion on the superior aspect of the patella with edematous type signal abnormality, which was hypointense on T1-weighted images hyperintense on FAT-SAT (Fig. 2). At that time, the differential diagnoses were chondroblastoma and osteoblastoma, which may overlap in their radiological appearance. The patient underwent an excisional biopsy and granular bone substitute was used with an immediate postoperative radiograph (Fig. 3). Pathological examination showed bony trabecula surrounded by numerous foamy enlarged histiocytes composed with round to oval hypochromatic nuclei and abundant eosinophilic cytoplasm, often containing engulfed inflammatory cells known as emperipolesis, admixed with a dense inflammatory mononuclear cells comprising lymphocytes, neutrophils, and scattered eosinophils and plasma cells (Fig. 4). Immunohistochemistry showed that the histiocytes were positive for S-100 protein and negative for CD-1a (Fig. 5). The conventional radiograph was reviewed and a slightly lytic lesion with a sclerotic rim was noticed in frontal section. A thoraco-abdominal pelvic scan was performed and showed no other distant localization. The constellation of these clinical and pathological findings led to the diagnosis of a solitary patellar RDD. The postoperative course was simple and 6 weeks postoperatively, the patient is clinically well. Control radiograph showed the beginning of integration and ossification of the bone substitute by the surrounding normal bone. Further scans and radiographs of the patient revealed no other lesion and no lymph node involvement. Given the patient's age and the fact the lesion was causing no pain or illness, no treatment was pursued. The patient will be followed every 6 months for 2 years with radiographic controls and a complete clinical examination.

The work has been reported in line with the SCARE 2020 criteria [8].

## 3 Discussion

This case describes the clinical, radiological and pathological course of an isolated patellar RDD.

Primary osseous involvement of RDD is rare, representing in roughly 10 % of cases and occurs in adulthood [4], [5]. The most primary osseous sites involved are the cranium (31 %), facial bones (22 %), and tibia (18 %) followed by the spine/sacrum, femur, and pelvis [1]. Primary involvement of the patella was described in one case of 12-year-old girl [7]. In the present case, RDD was diagnosed in the patellar with no other nodal or extranodal findings.

The imaging manifestations of RDD in bone are not specific. Bone lesions in RDD are typically osteolytic with margins varying from sclerotic to permeative on conventional radiographs [9], [10]. Purely sclerotic bone lesions are exceedingly rare. Aggressive features, like cortical destruction and associated soft-tissue mass are rarely present [6]. MRI features summarized by Hong and Jiang et al. [8], [9] fit well with our observation. Lesions are reported as well circumscribed and homogeneously image enhanced after gadolinium administration. They tend to be iso- or slightly hypointense on T1-weighted images and iso- or slightly hyperintense on T2-weighted images [11].

Numerous neoplastic and inflammatory benign diseases can overlap with RDD's imaging symptoms. The most prevalent diagnosed benign patellar tumor is the giant cell tumor (GCT). Imaging features of GCT are typical on radiographs including a pathologic fracture, a soap bubble appearance, a sclerotic and radiolucent lesion, a fracture of the femur and the tibia, and an osteolytic lesion of the patella with bone degradation. MRI shows an abnormal extension and lesion of the patella, and there may be some evidence of adjacent tissues and sclerotin [12]. The possibility of a GCT was not raised in our case as MRI features were not suggestive and the diagnosis was made on incisional biopsy. Osteolytic bone metastases are osteolytic lesions that cause thin and rupture of the bone cortex without causing a periosteal response [13]. In our case, a thoraco-abdominal pelvic scan was performed and showed no distant lesion. Thus, the possibility of a metastatic carcinoma was ruled out. As for infection, routine laboratory tests were within the normal ranges and there was no lymphadenopathy palpable clinically nor in thoraco-abdominal pelvic scan.

Histopathological examination with immunohistochemistry must be performed to confirm the final diagnosis of bone RDD. In our case, the patient underwent a biopsy and surgical resection. Microscopically, RDD is characterized by a proliferation of histiocytes that express S-100 protein and CD68 and are typically negative for CD1a [7]. The presence of inflammatory cells with a predominance of lymphocytes is also suggestive. A characteristic finding in RDD is emperipolesis defined by the presence of the engulfment of these inflammatory cells by the histiocytes [11]. In cases that reported the histomorphological features of the osseous RDD, classic features of nodal RDD were described. In some lesions, the large histiocytes were present focally, whereas in others they predominated. There is a greater tendency of the lesions to undergo fibrosis and less conspicuous emperipolesis. Other distinctive features are the presence of foamy macrophages, clusters of neutrophils resembling microabscesses, foci of fibrosis, and spindle cell areas with a storiform pattern. Eosinophils are rare and usually absent. Foci of necrosis with granuloma-like arrangement of histiocytes may be seen. The lesions demonstrate permeative growth with osteoclastic resorption of the preexisting trabecular bone. Reactive bone formation is common [6], [14].

Similar histiocytic diseases such as Langerhans cell histiocytosis and Erdheim-Chester disease can mimic RDD. Microscopic examination and immunohistochemistry help make the right diagnosis. Langherans cell histiocytosis is characterized by slightly foamy histiocytes with groovy nuclei mixed with inflammatory infiltrate containing eosinophils. Langherans cells express typically CD1a [15]. Erdheim-Chester disease is made of a diffuse proliferation of small histiocytes with moderate amount of cytoplasm in a fibrotic background. It is characterized by the absence of emperipolesis. Histiocytes express CD68, focally S-100 protein and does not express CD1a [15].

Treatment for primary RDD of bone is focused on relieving painful disease sites or avoiding consequences such pathologic fracture. Primary RDD of bone is not known to have a mortality risk. The most frequently mentioned therapies in these cases are surgical excision or curettage and bone grafting [10]. There is no established procedure for illness surveillance, and many patients are maintained with the expectation that symptoms will return or develop in novel ways. If this happens, more imaging will be done [10]. The prognosis is good to excellent with rare recurrences occuring in bone or lymph nodes [6].

## 4 Conclusion

This case presentation highlights the isolated patellar location of RDD. Clinical manifestations of patellar RDD are not specific. MRI imaging are adjunct techniques that could be helpful to guide the diagnosis but remain insufficient. Definitive diagnosis relies on the typical histopathological findings by the identification of the characteristic histiocytes showing emperipolesis expressing S-100 protein.

## Consent

Written informed consent was obtained from the patient for publication of this case report and accompanying images. A copy of the written consent is available for review by the Editor-in-Chief of this journal on request.

## Sources of funding

No.

## Provenance and peer review

Not commissioned, externally peer-reviewed.

## Credit authorship contribution statement

All the authors read and approved the final version of the manuscript.

Farah Sassi (MD): conception, acquisition of data, literature research and preparing the manuscript

Haythem M'rad (MD): acquisition of clinical data, preparing and revising the manuscript

Linda Belhaj Kacem (MD): Supervision

Boubaker Sassi (MD): revising the manuscript critically

Samia Hannachi (MD): acquisition of data, manuscript editing

Soumaya Rammeh (MD): final approval of the version to be published

## Guarantor

Sassi Farah

## Declaration of competing interest

The authors report no declarations of interest.
